# Discovery and design of photocyclic animal opsins: potential application to gene therapy from non-visual opsin research

**DOI:** 10.1186/s40662-025-00463-z

**Published:** 2025-11-02

**Authors:** Takahiro Yamashita

**Affiliations:** Department of Biophysics, Graduate School of Science, Kyoto University, Kyoto, 606-8502 Japan

## Abstract

Opsins are universal photoreceptive proteins in animals. Rhodopsin is the best-studied opsin and functions as a visual sensor in rod cells of human and mouse retinas. Rhodopsin produces an active state upon photoreception, which triggers the signal transduction cascade to evoke a hyperpolarizing response of the cells. This active state is a metastable intermediate and cannot convert back to the dark state by either photoreaction or thermal reaction. Thus, vertebrate rhodopsin is categorized as a mono-stable opsin. Recent accumulation of genomic information in animals has expanded the known repertoires of opsin genes, which are responsible for visual and non-visual photoreceptive functions. The analysis of these opsins revealed that many opsins, including non-visual opsins such as Opn4 and Opn5, form a stable active state upon photoreception and this active state can photo-convert back to the dark state. These opsins have the property of photoreversibility between the dark and active states and thus are categorized as bistable opsins. In addition, we previously identified a different type of non-visual opsin, Opn5L1, whose activity is controlled by a photocyclic reaction. This photocyclic reaction is quite similar to that of channelrhodopsin and is achieved by a special mechanism involving a cysteine residue at position 188 that has not been observed in any other opsins so far. This review would like to focus on the unique photocyclic animal opsin in the context of the diversity of visual and non-visual opsins and also discuss the possibility of designing “artificial photocyclic opsins” from natural opsins for potential application in optogenetic gene therapy.

## Background

Vision starts with the photoreception of visual cells in the retina. In general, vertebrate retina contains two types of visual cells, rod cells and cone cells [1, 2]. These cells are morphologically distinct and function under different light conditions. Rod cells work under dim light conditions for scotopic vision, whereas cone cells work under bright light conditions for photopic vision. These visual cells employ different types of photoreceptive protein opsins, namely rhodopsin for rod cells and cone visual pigments for cone cells. Rhodopsin is the historically best-studied opsin and has molecular properties important for visual photoreception. Recent accumulation of genomic information has unexpectedly expanded the repertoires of opsin genes in animals [3, 4]. It is well-known that many arthropod and mollusk species have characteristic eyes and utilize rhodopsin whose molecular properties are different from those of vertebrate rhodopsin for visual photoreception. Moreover, many vertebrates, including humans, have a variety of non-visual opsins in addition to visual opsins (rhodopsin and cone pigments). The molecular properties of visual and non-visual opsins from various animal species have been analyzed in order to understand the molecular basis of visual and non-visual functions. Recently, we identified a unique non-visual opsin from non-mammalian vertebrate species [5]. This non-visual opsin, Opn5L1, forms an active state in the dark and photo-converts to an inactive state which spontaneously reverts back to the original active state. Thus, the activity of this opsin is regulated by a photocyclic reaction. Such a photocyclic reaction has generally been found in microbial opsins such as channelrhodopsin [6], but has not been observed in animal opsins characterized so far. Moreover, based on the molecular mechanism of this photocyclic non-visual opsin, we successfully designed an “artificial mammalian rhodopsin” whose activity is regulated by a photocyclic reaction [7]. This photocyclic rhodopsin can be engineered from human rhodopsin. This review would like to show the diversity of the molecular properties of animal opsins and discuss the design of “artificial opsins” with potential application in optogenetic gene therapy.

## Main text

### Morphological characteristics of visual cells

**Fig. 1:**
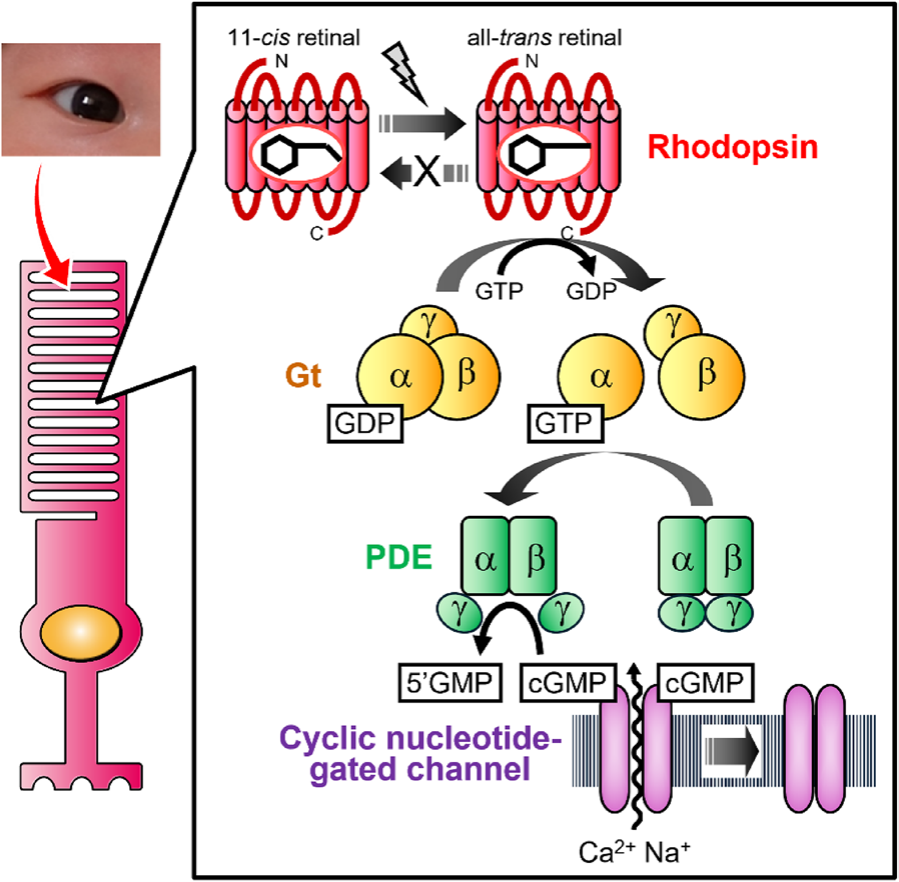
Schematic presentation of signal transduction cascade in a vertebrate rod cell. Rod cells have stacked disk membranes derived from the cilia that extend from the cell surface [8]. In this stacked membrane structure, mono-stable rhodopsin triggers a Gt-mediated signal transduction cascade to evoke a hyperpolarizing response [17]. PDE, phosphodiesterase; cGMP, cyclic guanosine monophosphate; 5’GMP, guanosine 5’-monophosphate

Many vertebrates, including humans, have visual cells in the retina that are neurons specialized for visual photoreception. These vertebrate visual cells are morphologically classified into two types: rod cells and cone cells [1, 2]. They function under different light conditions, with rod cells mainly responsible for scotopic vision and cone cells mainly responsible for photopic vision. These visual cells have a characteristic morphology and contain highly organized stacked membranes in their outer segments (Fig. 1). Rod cells have stacked disk membranes separated from the plasma membrane, whereas cone cells have a stack of membrane infoldings not separated from the plasma membrane. These stacked membrane structures contribute to the arrangement of a large fraction of the membrane protein opsins. Dense packing of photoreceptive protein opsins in the stacked membranes increases the efficiency of photon capture. This membrane structure in the outer segments of vertebrate visual cells is morphogenetically derived from the cilia that extend from the cell surface. Thus, vertebrate visual cells are called ciliary photoreceptor cells [8].

**Fig. 2:**
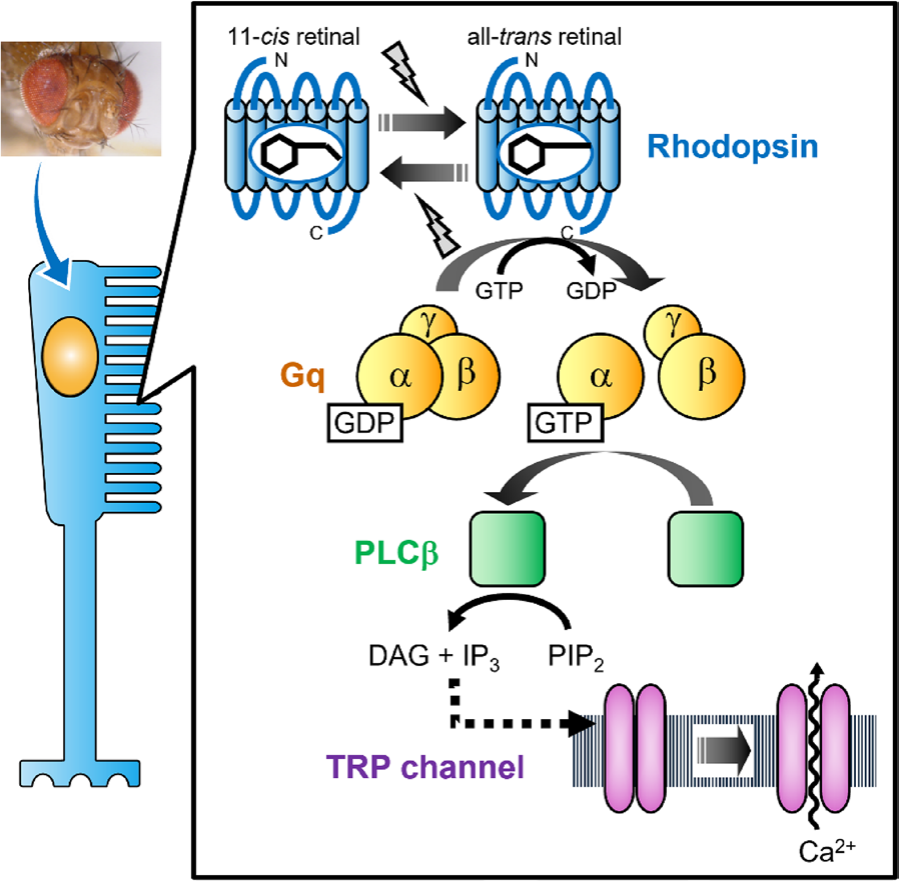
Schematic presentation of signal transduction cascade in Drosophila melanogaster visual cell. Insect visual cells have stacked membranes which consist of long, thin microvilli originating from the cell membrane [8]. In this stacked membrane structure, bistable rhodopsin triggers a Gq-mediated signal transduction cascade to evoke a depolarizing response [17]. PLC, phospholipase C; PIP_2_, phosphatidylinositole-4,5-bisphosphate; IP_3_, inositol-1,4,5-triphosphate; DAG, diacylglycerol; TRP, transient receptor potential

Like vertebrates, mollusks and arthropods have visual cells in their retinas for visual photoreception. These visual cells also have a stacked membrane structure that contains a large amount of opsin molecules to improve the efficiency of photon capture (Fig. 2). However, the membrane structure of mollusk and arthropod visual cells differs from that of vertebrate visual cells in morphogenesis. The membrane structure in the outer segments of mollusk and arthropod visual cells is an array of long, thin rhabdomeric microvilli originating from the cell membrane. Thus, mollusk and arthropod visual cells are called rhabdomeric photoreceptor cells [8]. The ciliary photoreceptor cells are responsible for vertebrate vision and have also been found in several protostome species such as scallop and annelid worm and non-bilaterian species such as jellyfish [9]. The rhabdomeric photoreceptors are mainly responsible for mollusk and arthropod vision and have also been found in several deuterostome species such as amphioxus and sea urchin [9]. Therefore, it can be speculated that two types of prototypical photoreceptor cells, “ciliary-type” and “rhabdomeric-type”, would have emerged in the common ancestor of animals [8, 10].

### Molecular properties of vertebrate rhodopsin and mollusk and arthropod rhodopsins

Vertebrate rhodopsin is the best-studied opsin; its analysis has a long history since it was first described in a paper about 150 years ago [11]. Rhodopsin has seven transmembrane helical domains which are a typical structural element of G protein-coupled receptors (GPCRs) [12]. To function as a photoreceptive protein, rhodopsin binds a chromophore, 11-*cis* retinal, through a Schiff base linkage to a lysine residue in Helix VII [1, 2]. Light induces the isomerization of the retinal from the 11-*cis* form into the all-*trans* form, which results in the formation of an active state of rhodopsin. This active state of rhodopsin interacts with the heterotrimeric G protein transducin (Gt) which is specifically expressed in vertebrate visual cells and facilitates the GDP-GTP exchange reaction on the α subunit of Gt. The α subunit of Gt then dissociates from the βγ subunit complex and stimulates phosphodiesterase (PDE) to hydrolyze cyclic guanosine monophosphate (cGMP). Then, the cyclic nucleotide-gated channels in the plasma membranes of rod cells, which are open in dark conditions, close due to a decrease of cGMP concentration in light conditions. The closure of the channels eventually evokes the hyperpolarization of the cells [13] (Fig. 1). Thus, the electrical response of vertebrate “ciliary-type” visual cells occurs through the intracellular cyclic nucleotide signaling triggered by the photoactivation of rhodopsin. The detailed molecular properties of vertebrate rhodopsin have been analyzed by biochemical and biophysical methods [1, 2]. In vertebrate rod cells, rhodopsin molecules simultaneously photo-convert to active states. This active state is thermally unstable and spontaneously releases all-*trans* retinal to form an apo-protein. This apo-protein takes up 11-*cis* retinal, which is supplied from the visual cycle mainly in the retinal pigment epithelium, and regenerates the original dark state (11-*cis* retinal bound state). Thus, the assistance of the 11-*cis* retinal supplying system is essential for the formation of the dark state (inactive state) from the active state, and a thermal reaction or a second photon absorption cannot lead to the direct conversion from the active state to the dark state. In addition, the active state cannot be formed by the direct incorporation of all-*trans* retinal into an apo-protein. Therefore, vertebrate rhodopsin is specialized for photoreception and is categorized as a mono-stable opsin because only the dark state is thermally stable [14] (Fig. 1).

Mollusk and arthropod rhodopsins also have common structural elements, including seven transmembrane domains and a chromophore, 11-*cis* retinal [15]. After photoreception, mollusk and arthropod rhodopsins form an active state by the isomerization of the retinal to the all-*trans* form. This active state of rhodopsin activates Gq-type of G protein to stimulate phospholipase Cβ (PLCβ). PLCβ then catalyzes the conversion of phosphatidylinositole-4,5-bisphosphate (PIP2) to inositol-1,4,5-triphosphate (IP_3_) and diacylglycerol (DAG). Finally, the light-dependent opening of Ca^2+^ channels, which are known as transient receptor potential (TRP) and TRP-like (TRPL) channels in *Drosophila melanogaster*, in the plasma membrane evokes the depolarization of visual cells, although the detailed molecular components linking PLCβ activation and Ca^2+^ channel opening are still controversial [16]. Thus, the electrical response of mollusk and arthropod “rhabdomeric-type” visual cells is thought to occur through the intracellular IP_3_/Ca^2+^ signaling triggered by the photoactivation of rhodopsin [17] (Fig. 2). The analysis of the molecular properties of mollusk and arthropod rhodopsins clearly highlights several differences from the molecular properties of vertebrate rhodopsin [14]. After capturing photons, mollusk and arthropod rhodopsins form thermally stable active states (all-*trans* retinal bound state) which do not immediately release all-*trans* retinal. After subsequently capturing photons, this active state can return to the original dark state (11-*cis* retinal bound state). This means that light can efficiently facilitate the isomerization of the retinal from the all-*trans* form to the 11-*cis* form within mollusk and arthropod rhodopsins, which is not observed in vertebrate rhodopsin. Thus, mollusk and arthropod rhodopsins have two stable states, the dark state (inactive state) and active state, which can be converted to each other by photoreception. In addition, the apo-proteins of mollusk and arthropod rhodopsins can directly incorporate all-*trans* retinal to form active states without light signals [14]. This is a common observation in many GPCRs that can be activated by the direct binding of diffusible ligands known as agonists, but is not observed in vertebrate rhodopsin. Based on these molecular properties, mollusk and arthropod rhodopsins are categorized as bistable opsins which have thermally stable dark and active states [14] (Fig. 2).

### Molecular diversity of opsins responsible for “non-visual” photoreception

Animals also utilize light from the environment as various information sources for physiological functions other than vision [18–20]. Humans unconsciously adjust the pupil’s size in response to light. This contributes to adaptation to different light conditions and is known as the pupillary light reflex. Many animals, including humans, know time based on changes in their light environment. This is achieved by the entrainment of their internal circadian clocks by light signals. In addition to changes in behavior based on circadian rhythms, some animals change their behaviors in response to seasonal changes, such as reproductive behavior during certain seasons. These behaviors are triggered by the perception of changes in day length, which is known as photoperiodism. Moreover, some animals change their body color to match the surrounding light environment. This is known as background adaptation and helps the animals blend in with their surrounding environment to avoid detection by predators. These light-dependent physiological functions are called “non-visual” photoreceptive functions, most of which are considered to be triggered by photoreception by opsins. Recent advances in genome analysis in animals have improved our understanding of the molecular entities underlying non-visual photoreceptive functions. Humans have four visual opsin genes, rhodopsin and three cone pigments [21]. The three cone pigments each have their characteristic spectral sensitivity, that is, red-, green- and blue-sensitive ones, which contrasts with the sole rod pigment, green-sensitive rhodopsin. This repertoire of visual opsins leads to color vision under bright light conditions and monochromatic vision under dim light conditions. Moreover, it has been revealed that the human genome contains additional five opsin genes, *OPN3*, *OPN4*, *OPN5*, *RGR* and *RRH*, which are thought to contribute to non-visual photoreceptions [22].

Among these non-visual opsins, retinal G protein-coupled receptor (RGR) was the first identified opsin [23]. RGR protein is mainly distributed in the retinal pigment epithelium. The protein exclusively binds all-*trans* retinal, not 11-*cis* retinal, in the dark and generates 11-*cis* retinal by isomerization of the retinal after photoreception [24]. However, the protein is thought to lack the ability to activate G protein. Analysis of RGR-deficient mice shows the physiological role of RGR as a retinal photoisomerase that supplies 11-*cis* retinal to visual opsins [25]. Thus, although RGR belongs to the GPCR family, the opsin has an important molecular function other than G protein activation.

**Fig. 3:**
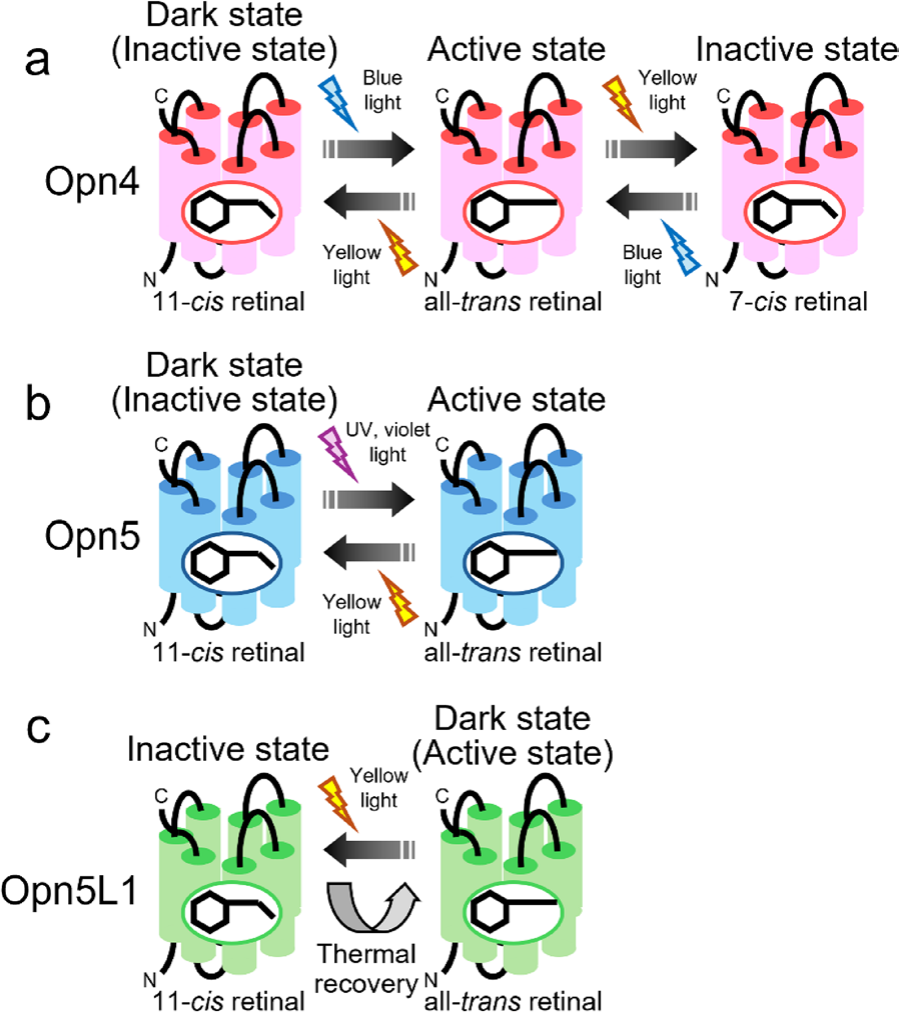
Diversity of the molecular properties of non-visual opsins. **a** Mammalian Opn4 is a tristable opsin, a kind of bistable opsin, that exhibits photo-equilibration among two inactive states and an active state [29, 30]. **b** Opn5 is a bistable opsin that exhibits photo-equilibration between the dark (inactive state) and active states [44]. **c** Opn5L1 has an active state in the dark and photo-converts to an inactive state, which thermally reverts to the active state (dark state) [5]. Hence, Opn5L1 is categorized as a photocyclic opsin

Opn4 is the best-studied non-visual opsin among vertebrate and invertebrate non-visual opsins characterized so far. Opn4 was originally isolated from dermal melanophores of African clawed frog (*Xenopus laevis*), although it was also identified in the eyes and brain in this animal [26]. Its expression in the dermal melanophores suggests the possibility that Opn4 controls skin pigmentation related to body color change [27]. Thus, this opsin was named melanopsin. Since then, the discovery of its mammalian orthologue and its expression in a subset of ganglion cells in mammalian retina, called intrinsically photosensitive retinal ganglion cells (ipRGC), has boosted the understanding of the molecular properties and physiological relevance of Opn4 protein [28]. Opn4 protein binds 11-*cis* retinal to form a blue-sensitive pigment in the dark [29]. After photoreception, the isomerization of the retinal to the all-*trans* form induces the formation of an active state of Opn4 protein. This active state is thermally stable and photo-converts to not only the dark state (11-*cis* retinal bound state) but also the 7-*cis* retinal bound state. This 7-*cis* retinal bound state is another inactive state with blue-shifted spectral sensitivity and is converted to an active state by photoreception. This observation means that mammalian Opn4 exhibits unique photo-equilibration among two inactive states and an active state, which can temporally and chromatically broaden the tuning of light-dependent activities in Opn4-expressing ipRGCs [30]. Therefore, mammalian Opn4 is a “tristable” opsin, a kind of bistable opsin like mollusk and arthropod rhodopsins (Fig. 3a). Moreover, in ipRGCs, Opn4 protein activates a Gq-mediated signal transduction cascade to evoke a cellular electrical response [31], which is also similar to the signaling in the rhabdomeric-type visual cells of mollusk and arthropod retinas. Analysis of *Opn4* knock-out mice has revealed several non-visual functions regulated by Opn4. The most important finding is the Opn4-dependent entrainment of the circadian rhythm. Although rod-less and cone-less mice still exhibited light-dependent modulation of the phase of the circadian oscillation [32], additional *Opn4* knock-out caused complete loss of the photoentrainment ability [33, 34]. However, *Opn4* knock-out mice with functional rod and cone cells maintained the photoentrainment of the circadian rhythm [35, 36]. These results indicate that Opn4 in ipRGCs has a functional redundancy with rhodopsin and cone pigments in photoentrainment of the circadian oscillation [37]. In addition, the pupillary light reflex is also under the control of Opn4, which is redundant with the contribution of rhodopsin and cone pigments [38]. It is now clear that photoreception via Opn4 in ipRGCs can regulate other important non-visual functions in the eyes and brain [39–42].

### Discovery of photocyclic animal opsin from non-visual opsin research

Among the human opsin genes, *Opn5* is the most recently identified. The first report about Opn5 (called neuropsin) in 2003 revealed the expression of Opn5 mRNA in the retina and brain [43]. However, for some time thereafter, there were no reports on the molecular properties, detailed expression patterns and physiological functions of Opn5 protein. In this context, we reported the detailed molecular properties of Opn5 protein in the early 2010s [44, 45]. Opn5 protein directly binds 11-*cis* retinal to form a short wavelength-sensitive pigment whose spectral sensitivity covers from the ultraviolet (UV) region to the blue region. This spectral sensitivity is conserved among Opn5 proteins of a wide range of vertebrate species from fishes to human. This means that Opn5 protein provides one of the molecular bases underlying the absorption of the shortest wavelength light in vertebrates, including humans. After photoreception, Opn5 protein forms an active state by the isomerization of the retinal to the all-*trans* form. This active state is thermally stable and can convert back to the original dark state upon subsequent photoreception. Hence, Opn5 is categorized as a bistable opsin (Fig. 3b). We also analyzed the detailed expression patterns of Opn5 mRNA or protein in the retina and brain of mouse and common marmoset (a species of New World monkey) [45]. We detected Opn5-expression signals in a subset of the ganglion cells in the retina and the preoptic area in the hypothalamus, which were conserved between mouse and common marmoset. The analyses of the recombinant proteins of mammalian Opn5 and the cultured cells expressing mammalian Opn5 showed that mammalian Opn5 protein can activate Gi-type or Gq-type of G protein [44, 46–48]. However, there are no studies that have shown light-dependent electrophysiological responses from Opn5-positive cells in the mouse retina or brain, probably because of the low Opn5 protein expression level in these cells and a lack of suitable mouse lines for visualizing Opn5-positive cells. Since the late 2010s, the physiological functions of Opn5 have been reported based on analyses of *Opn5* knock-out mice. Opn5-deficient mice maintain normal electroretinograms under dark- and light-adapted conditions [49]. However, *Opn5* knock-out leads to a loss of synchronization of local circadian oscillators in the retina, cornea, and skin to light/dark cycles and also impairs circadian photoentrainment of behavior under short wavelength conditions [49–52]. Thus, the Opn5-dependent reset of the circadian oscillator in the retina may affect the behavioral rhythm. As mentioned above, Opn5 protein has a relatively broad absorption spectrum which can cover an expanded wavelength range from the UV region to the blue region [44]. This spectral characteristic is not suitable for visual color discrimination but is possibly advantageous for resetting circadian rhythm. Moreover, Opn5 mediates light-dependent vascular development in the retina and vitreous body [53]. Therefore, short wavelength light reception via Opn5 works as an important developmental timing cue to regulate optic axis clearance for normal visual function. Opn5 also regulates choroidal thickness in the eyes, which plays a key role in adjusting the size of the eyeball [54]. This means that exposure to short wavelength light for a certain period each day can prevent elongation of the eyeball to suppress myopia. In addition to mounting evidence reporting important roles of Opn5 in the retina, the physiological relevance of Opn5 in the brain has been revealed through studying knock-out mice. A subset of Opn5-positive cells in the preoptic area project to brown adipose tissue to regulate thermogenesis [55]. Although it has been controversial whether mammals can detect light signals in the brain for some physiological functions, this was the first paper that clarified an opsin-mediated light-sensing pathway in the mammalian brain. The functional importance of short wavelength-sensitive Opn5 protein has also been reported in non-mammalian vertebrates. In avian species, Opn5 is distributed in a subset of amacrine and ganglion cells in the retina, the pineal gland and the paraventricular organ of the hypothalamus [44, 56]. Opn5-expressing cells in the pineal gland exhibit serotonin-positive signals, suggesting that these cells are involved in melatonin secretion. Opn5-expressing cells in the paraventricular organ also overlap with serotonin-positive signals, suggesting that these cells work as photoperiodic sensors to induce testicular growth under long-day conditions. In medaka fish, Opn5-positive cells are uniquely observed in the pituitary gland and release melanocyte-stimulating hormone under short wavelength conditions to induce black pigmentation in the body [57, 58]. This mechanism may contribute to protection from UV light.

**Fig. 4:**
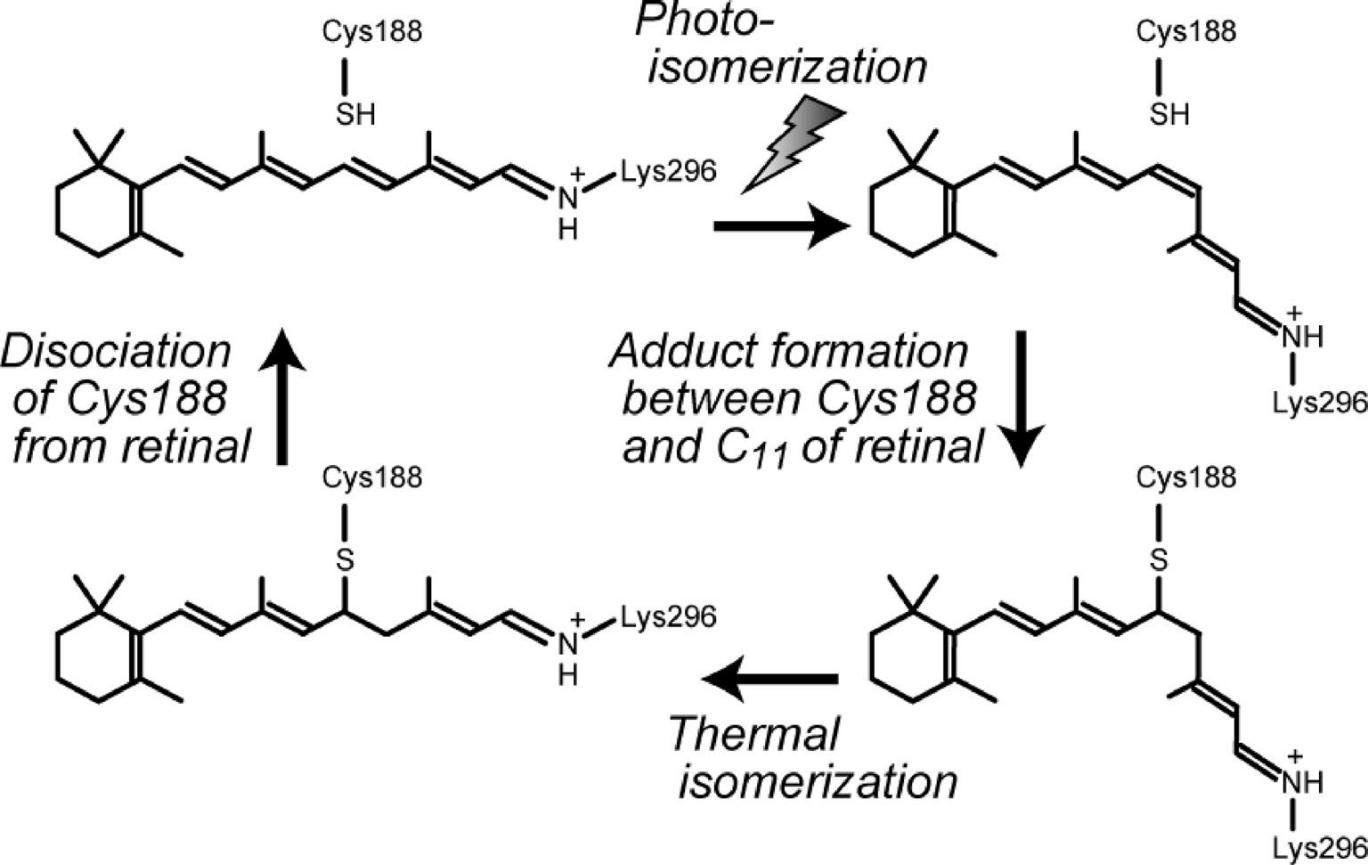
Light-triggered conformational change of the retinal within the Opn5L1 protein. Opn5L1 binds all-*trans* retinal to form an active state in the dark [5]. Light causes retinal isomerization to the 11-*cis* form to suppress the activity of Opn5L1. In this state, Cys188 and C11 of retinal form an adduct, thereby converting the double bond of C11=C12 to a single bond. Subsequently, thermal rotation of the C11–C12 single bond easily occurs to permit conversion to the all-*trans* form. In this state, Cys188 and C11 of the retinal dissociate to regenerate the dark state containing all-*trans* retinal

Searches for Opn5 genes in vertebrate genomes have shown that mammals, including humans and mice, have a single Opn5 gene, whereas non-mammalian vertebrates have additional Opn5 genes, such as *Opn5L1* and *Opn5L2*, which are paralogous to mammalian Opn5 [22, 59, 60]. Our analysis of these Opn5 genes found in non-mammalian vertebrates revealed that Opn5L1 is a unique photocyclic opsin whose molecular properties are quite different from those of mono-stable and bistable opsins (Fig. 3c) [5]. In the chicken retina, Opn5L1 is expressed in the inner side of the inner nuclear layer, possibly a subpopulation of amacrine cells. Opn5L1 is also distributed in multiple regions of the chicken brain, including the mesopallium in the telencephalon and the paraventricular nucleus in the diencephalon. Thus, Opn5L1 is considered to function as a non-visual opsin in the retina and brain of non-mammalian vertebrates. Comparison of the amino acid sequences among vertebrate Opn5 proteins shows that Opn5L1 proteins have characteristic mutations in the triad motif Asp-Arg-Tyr (DRY) on the cytoplasmic side of Helix III. This triad motif is well conserved among rhodopsin-like GPCRs, including human and mouse Opn5 proteins, and regulates GPCR conformational changes [61]. However, for example, chicken Opn5L1 protein contains Val-Cys-Cys (VCC) in place of the DRY motif. This suggested the possibility that Opn5L1 proteins are deficient in G protein activation ability. In this context, we analyzed the detailed molecular properties, that is, the binding preference of retinal isomers, photoactivation process and G protein activation, of Opn5L1 protein. Regarding the binding selectivity toward retinal isomers, Opn5L1 protein bound all-*trans* retinal even after it had been incubated with 11-*cis* retinal. This means that Opn5L1 protein exclusively binds all-*trans* retinal and has lost the ability to directly incorporate 11-*cis* retinal. Our spectral analysis showed that chicken Opn5L1 protein incorporates all-*trans* retinal to form a green-sensitive pigment whose spectral peak is located at 510 nm. We also observed that this direct binding of all-*trans* retinal to the apo-protein of Opn5L1 resulted in a significant increase of G protein activation ability despite mutations in the conserved triad DRY motif on the cytoplasmic side of Helix III. Hence, all-*trans* retinal can function as an agonist to form an active state of Opn5L1 protein without light signals. However, it should be noted that the addition of other all-*trans* retinoids, such as retinol and retinoic acid, to Opn5L1 protein cannot induce the G protein activation. We also analyzed the light-dependent changes of the G protein activation ability and the absorption spectrum of chicken Opn5L1 protein. The G protein activation ability of Opn5L1 protein in the dark was suppressed by yellow light irradiation, which indicates that Opn5L1 protein can be deactivated by light signals. In parallel, light irradiation caused the complete elimination of absorbance in the visible and near-UV regions and a small increase of absorbance at 270 nm. Formation of the “270 nm product” has not been observed with other opsins and is thought to originate from an unknown mechanism other than retinal photo-isomerization. Our detailed analysis unveiled that light signals induce isomerization of the retinal from the all-*trans* form to the 11-*cis* form to produce an intermediate state whose spectral peak is located at 500 nm, which is followed by the formation of the “270 nm product” by an unknown thermal reaction. It should be noted that the formation of the “270 nm product” does not occur by the release of 11-*cis* retinal after the hydrolysis of the Schiff base linkage. Moreover, we observed subsequent changes of the G protein activation ability and the absorption spectrum of Opn5L1 protein. We detected recovery of the G protein activation ability during incubation in the dark. This increase of the activity is coupled with the recovery of absorbance at 510 nm in the absorption spectrum. This means that the “270 nm product” can undergo thermal reversion to the original dark state (all-*trans* retinal bound state). To gain insight into this characteristic molecular property of Opn5L1 protein, we searched for the amino acid residue(s) responsible for this unknown thermal reaction. We successfully identified a key cysteine residue at position 188, which is well conserved among Opn5L1 proteins of non-mammalian vertebrates and not in other Opn5 proteins. After photoreception, the C188T mutant of Opn5L1 protein forms an intermediate state, that is, the “500 nm product”, but subsequently cannot convert to the “270 nm product”. This shows that the unique formation of the “270 nm product” depends on some molecular mechanism involving Cys188. Then, we hypothesized that, after photoreception of Opn5L1 protein, the retinal-conjugated double bond system is disrupted by the formation of a covalent adduct between retinal and the cysteine residue. Liquid chromatography-mass spectrometry analysis confirmed that a short peptide fragment containing Cys188, which is formed by proteolysis of Opn5L1 protein, binds to the retinal in the “270 nm product”. This result supports the hypothesis that an adduct is formed between Cys188 and retinal in the “270 nm product”. Based on our experimental data, the predicted mechanism underlying the molecular function of Opn5L1 is as follows (Fig. 4):

1. The dark state exclusively binds all-*trans* retinal to form an active state. After photoreception, the retinal is isomerized to the 11-*cis* form to suppress the G protein activation ability.
2. Cys188 approaches C11 of retinal to form a covalent adduct, which induces conversion of the C11=C12 double bond in retinal to a single bond.
3. The C11–C12 single bond in the retinal undergoes thermal rotation.
4. The Cys188-retinal adduct dissociates in a *trans* conformation, which regenerates the original dark state (active state) to recover the G protein activation ability.

It is well known that light-oxygen-voltage proteins, which are widespread in plants and microbes, form an adduct between their chromophore, flavin, and an adjacent cysteine residue after photoreception [62]. Among the opsins characterized so far, the formation of an adduct between the retinal and the cysteine residue is uniquely observed in Opn5L1 protein. However, a molecular model in which an adduct formation between the retinoid and the cysteine residue can facilitate the thermal isomerization of the retinoid has previously been proposed in RPE65 protein [63]. RPE65 protein works as a retinoid isomerohydrolase to catalyze the conversion of all-*trans* retinyl esters to 11-*cis* retinol in the retinal pigment epithelium [64]. Thus, RPE65 protein is a key component of the visual cycle and one of the important targets for gene therapy such as Luxturna [65]. Recent structural analysis of RPE65 protein revealed that there are no appropriate cysteine residues in the retinoid binding pocket of RPE65 protein and a covalent bond between a cysteine and a retinoid is not formed in RPE65 protein [66]. Our analysis revived a classical molecular model involving the formation of an adduct, which explained the thermal isomerization of the retinal in Opn5L1 protein.

The Opn5L1 gene can be found in a wide range of non-mammalian vertebrate species and Opn5L1 protein has characteristic molecular properties different from those of mono-stable opsins such as vertebrate rhodopsin and bistable opsins such as invertebrate rhodopsin and mammalian Opn5 [5]. Opn5L1 protein exclusively binds all-*trans* retinal, like mammalian RGR protein. However, Opn5L1 protein exhibits G protein activation ability under dark conditions and showed decreased ability under light conditions, which contrasts with mammalian RGR protein (that is thought to lack G protein activation ability). In addition, after photoreception, Opn5L1 can thermally self-regenerate to the dark state by thermal isomerization of the retinal. The combination of photoisomerization and thermal isomerization of retinal regulates the G protein activation ability of Opn5L1 protein, making this protein the first animal opsin whose activity is controlled by its photocyclic reaction. However, the physiological relevance of Opn5L1 protein remains unknown. Opn5L1 rapidly loses its activity under light conditions and gradually recovers its activity as darkness falls. Thus, Opn5L1 may be used as a timer to measure time elapsed since the last illumination, such as after sunset.

### Design of artificial photocyclic animal opsins

**Fig. 5:**
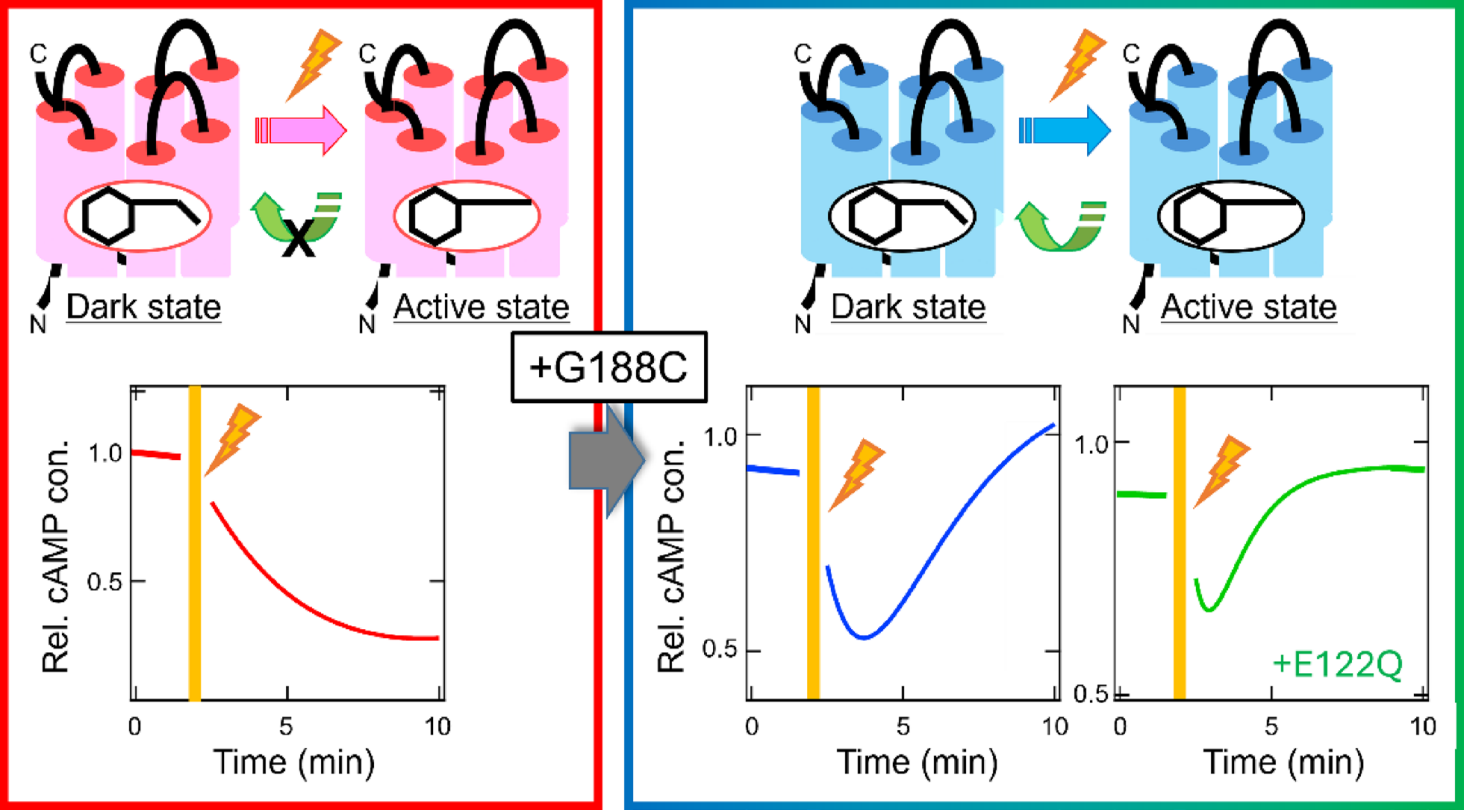
Creation of photocyclic vertebrate rhodopsin by a single mutation. After photoreception, vertebrate rhodopsin forms an active state with a certain lifetime and cannot thermally recover to the dark state (inactive state), which leads to a light-dependent prolonged decrease of the cAMP level by the activation of Gi-type of G protein in vertebrate rhodopsin-expressing mammalian cultured cells. Introduction of the G188C mutation enables vertebrate rhodopsin to acquire the ability to thermally convert to the dark state from the active state [7]. This photocyclic property shortens the lifetime of the active state and allows rapid recovery of the cAMP level after light-induced decrease in mutant rhodopsin-expressing cells. Introduction of the additional mutation E122Q into the photocyclic mutant rhodopsin further shortens the lifetime of the active state, which leads to a transient light-induced decrease in the cAMP level in mutant rhodopsin-expressing cells. cAMP, cyclic adenosine monophosphate

Opn5L1 protein photo-converts to an inactive state which is thermally unstable and spontaneously reverts back to the original state. This property contrasts with the property of bistable opsins, that is, bistable opsins have two stable states, a dark state (inactive state) and an active state, which can photo-convert with each other. At present, Opn5L1 protein is the only animal opsin whose activity is known to be controlled by photocyclic reaction. This unique photocyclic reaction is achieved by a characteristic cysteine residue at position 188, which is conserved among Opn5L1 proteins and rarely found in other opsins. In this context, we next tried to create artificial photocyclic opsins by the introduction of a cysteine residue into position 188 of other opsins. First, we introduced this mutation at position 188 of vertebrate UV-sensitive bistable opsin Opn5 protein [67]. T188C mutant of Opn5 protein exclusively binds all-*trans* retinal and loses the ability to directly incorporate 11-*cis* retinal. This alteration of the binding preference of the retinal isomers in the Opn5 mutant protein mimics the property of Opn5L1 protein. In addition, the T188C mutant of Opn5 protein produces a blue-sensitive pigment through the incorporation of all-*trans* retinal and forms a UV-sensitive state by the isomerization of the retinal to the *cis* forms after photoreception. Subsequently, during incubation in the dark, this state spontaneously reverts back to the original dark state by the thermal isomerization of the retinal to the all-*trans* form. The observation of this thermal recovery to the dark state means the acquisition of the photocyclic reaction of this mutant, like that of Opn5L1 protein. It should be noted that replacement of the residue at position 188 with a residue other than cysteine does not result in the acquisition of a photocyclic reaction. Thus, the introduction of the cysteine residue can dramatically convert vertebrate bistable opsin Opn5 into a photocyclic opsin. Second, we introduced the cysteine residue at position 188 of mammalian rhodopsin [7]. The G188C mutant of mammalian rhodopsin binds 11-*cis* retinal to form a blue-sensitive pigment whose spectral peak is blue-shifted from that of the original green-sensitive pigment. After photoreception of this mutant, the isomerization of the retinal to the all-*trans* form induces the formation of an active state whose spectral peak is in the UV region, like that of the original pigment. During incubation in the dark, this active state spontaneously reverts back to the dark state by the thermal isomerization of the retinal to the 11-*cis* form. This thermal recovery of the dark state is not observed in the original mammalian rhodopsin. Also, replacement of the residue at position 188 with a residue other than cysteine does not result in the acquisition of this thermal recovery. Hence, the introduction of the cysteine residue can also dramatically convert mono-stable mammalian rhodopsin into a photocyclic opsin. It should be noted that the molecular mechanism underlying the acquisition of a photocyclic reaction in G188C mutant is unclear, because the formation of an adduct involving the introduced cysteine residue was not clearly observed in this mutant. The acquisition of the photocyclic reaction of mammalian rhodopsin by this mutation can be confirmed by the changes of the G protein activation profiles in mammalian cultured cells (Fig. 5). Mammalian rhodopsin can activate Gi-type of G protein in cultured cells to decrease the level of cyclic adenosine monophosphate (cAMP) in a light-dependent manner. In this case, a prolonged decrease in cAMP level was observed, reflecting the lifetime of the active state of rhodopsin. By contrast, the G188C mutant induced a rapid recovery of the cAMP level after the decrease of the level by light, which can be explained by the thermal recovery of the dark state (inactive state) from the photo-activated state. Introduction of the additional mutation E122Q to shorten the lifetime of the active state also induced a more rapid recovery from the decrease of the cAMP level. These results suggest the possibility that artificial photocyclic opsins can be designed by utilizing appropriate mutations of mammalian rhodopsin protein.

### Potential application of photocyclic opsins to optogenetic gene therapy

Photoreceptive proteins are responsible for various photo-responses in unicellular and multicellular organisms including bacteria, plants and animals. In the last two decades of research, photoreceptive proteins have been utilized to manipulate cellular functions by light. This technique, optogenetics, was chosen as Method of the Year 2010 by Nature Methods [68] and has been widely utilized to clearly demonstrate the relationship between cellular responses and physiological phenomena [69]. Optogenetics began with the discovery of channelrhodopsin, a light-gated cation channel, in a green alga [70]. Channelrhodopsin binds all-*trans* retinal as a chromophore and belongs to the microbial rhodopsin group. Thus, channelrhodopsin and animal opsins have quite different amino acid sequences and molecular functions [6]. Channelrhodopsin has several important molecular properties that can be utilized as an optogenetics tool, one of which is that its activity can be controlled by a photocyclic reaction. This photocyclic property results in repetitive and short-lived photoexcitation of channelrhodopsin-expressing neurons [71, 72]. By contrast, animal opsins have the potential to regulate intracellular G protein signaling by light. However, mammalian rhodopsin, a prototypical animal opsin, cannot spontaneously revert back to the dark state after photoactivation, which prevents repeated activation of G protein signaling by light [73]. As an alternative, bistable opsins, which are activated and inactivated by selective light irradiations, have been attracting attention as optogenetics tools [74, 75]. Moreover, artificial photocyclic opsins designed from mammalian rhodopsin can be advantageously utilized as optogenetics tools. Quite recently, optogenetics has begun to be applied to gene therapy. Introduction of a channelrhodopsin variant into retinal ganglion cells of a blind patient with retinitis pigmentosa partially restored visual function [76]. However, the acquired visual sensitivity was not high and there is room for improvement of the optogenetic gene therapy method. Experiments with mice have shown that ectopic expression of human rhodopsin in ON-bipolar cells of mice with retinal degeneration resulted in the recovery of visual function [77]. Photocyclic opsins designed from mammalian rhodopsin can be repeatedly activated by light without releasing all-*trans* retinal (of which accumulation in the tissues can be toxic [78]). Therefore, the application of these photocyclic opsins could be an alternative that would more significantly improve visual sensitivity while reducing damage to the retina.

## Conclusion

Advances in genome analysis have revealed that animals have a greater diversity of opsin genes than previously expected. Analysis of these opsins has unveiled the existence of non-visual opsins with diverse molecular properties and distribution patterns, which are thought to be the molecular basis for various photoreceptive functions other than vision. From the viewpoint of the diversity of molecular properties of visual and non-visual opsins, this review outlined the discovery of photocyclic opsin, which has not been found in animals. Furthermore, the discovery of photocyclic opsin has led to the creation of artificial photocyclic opsins based on mammalian rhodopsin. This artificial photocyclic opsin can be engineered from human rhodopsin, offering several advantages for use in optogenetic gene therapy. For example, this photocyclic opsin could be engineered from human rhodopsin originally possessed by humans, which could potentially evade the immune system when it is introduced into the human body. Moreover, the use of human rhodopsin-based photocyclic opsins could lower the psychological barriers for patients to undergo gene therapy compared to introducing genes from other organisms, such as green algae. Therefore, it is expected that combining the design of artificial photocyclic opsins with existing technologies and knowledge will contribute to the development of novel optogenetic gene therapy methods.

## Data Availability

Not applicable.
